# Workgroup Report: Base Stations and Wireless Networks—Radiofrequency (RF) Exposures and Health Consequences

**DOI:** 10.1289/ehp.9633

**Published:** 2006-11-06

**Authors:** Peter A. Valberg, T. Emilie van Deventer, Michael H. Repacholi

**Affiliations:** 1 Gradient Corporation, Cambridge, Massachusetts, USA; 2 Radiation and Environmental Health, World Health Organization, Geneva, Switzerland

**Keywords:** adverse health effects, cell telephones, electromagnetic waves, mechanisms, mobile telephony, nonionizing, RF modulation

## Abstract

Radiofrequency (RF) waves have long been used for different types of information exchange via the airwaves—wireless Morse code, radio, television, and wireless telephony (i.e., construction and operation of telephones or telephonic systems). Increasingly larger numbers of people rely on mobile telephone technology, and health concerns about the associated RF exposure have been raised, particularly because the mobile phone handset operates in close proximity to the human body, and also because large numbers of base station antennas are required to provide widespread availability of service to large populations. The World Health Organization convened an expert workshop to discuss the current state of cellular-telephone health issues, and this article brings together several of the key points that were addressed. The possibility of RF health effects has been investigated in epidemiology studies of cellular telephone users and workers in RF occupations, in experiments with animals exposed to cell-phone RF, and via biophysical consideration of cell-phone RF electric-field intensity and the effect of RF modulation schemes. As summarized here, these separate avenues of scientific investigation provide little support for adverse health effects arising from RF exposure at levels below current international standards. Moreover, radio and television broadcast waves have exposed populations to RF for > 50 years with little evidence of deleterious health consequences. Despite unavoidable uncertainty, current scientific data are consistent with the conclusion that public exposures to permissible RF levels from mobile telephony and base stations are not likely to adversely affect human health.

A vast number of communication networks interconnect societies worldwide, and cellular wireless technology networks make up an increasing fraction of this number. The presence of radiofrequency (RF) waves from wireless technologies has become ubiquitous. Mobile telephony (construction and operation of telephones or telephonic systems) is relied on by > 1.4 billion people, or around 20% of the world’s population. Given that the public is frequently reminded that we are all surrounded by ever-present electromagnetic fields (EMFs), which some call “electro-smog,” it is not surprising that some individuals and groups express concern about possible health effects from low-level, chronic exposure to a variety of RF sources. To help address this concern, the World Health Organization (WHO) convened a “Workshop on Base Stations and Wireless Networks” as part of its “International EMF Project” to discuss the state of the science in RF health effects. In this article we provide a summary of several key points addressed at the workshop.

RF waves have long been used for different types of wireless broadcast, such as for wireless Morse code, radio, television, and so on. The radio-wave spectrum spans the frequency range from about 0.5 MHz in the AM radio band up to about 30,000 MHz in the radar band. RF-emitting devices have become commonplace in homes, offices, and schools. Table 1 lists examples of RF sources contributing to the radio-wave background in almost every modern-day location. The actual RF level from each source depends on the details of the exposure location (i.e., distance from the antenna), but the last column lists whether the source is more ubiquitous and universal (U) or more limited and local (L); a “+” indicates that this source would typically contribute a significant fraction of ambient RF background levels. Even for individuals in the vicinity of transmitting antennas, surveys of RF levels report results that are far below the applicable exposure guidelines both in the United States (Burch et al. 2006; Tell and Mantiply 1980) and in Europe (Foster K, in press).

In addition to the increasing prevalence of cellular telephones, there has been continuing expansion of wireless Internet access, such as WiFi, into homes, schools, workplaces, and public areas. “WiFi” is an abbreviation for “Wireless Fidelity,” and is used generically when referring to any type of wireless technology that supports local, over-the-air computer communication via a wireless local-area network (WLAN). Typically, the transmission frequency is approximately 2.4 GHz, and WiFi provides data-transmission rates in the range of 1–50 Mbps (megabytes per second). “WiMAX” is a long-range version of WiFi. Cellular wireless technology is now capable of delivering voice, text, images, music, and other data to consumers everywhere, and it relies on an extensive network of fixed antennas, or base stations, for relaying information using RF signals. The number of base stations required increases with greater mobile phone use (requiring extensive micro-cell or pico-cell distributed antenna systems in urban areas), with market competition (enabling more operators to provide services), and with new technological capabilities (e.g., 3G). 3G (or 3-G) is short for “third-generation” mobile telephone technology. The services associated with 3G provide the ability to transfer both voice data (a telephone call) and nonvoice data (e.g., downloading information, exchanging email, and instant messaging).

The public, regulators, and scientists have questioned whether there are possible health consequences of this mushrooming mobile phone technology, particularly because the handset operates in close proximity to the human body and because large numbers of base station antennas are required. Although the RF levels produced by base stations at consumer locations are much lower than those from use of the phone handset, the more continuous exposure from base stations has produced a greater public concern.

## RF Exposure Levels

The total electromagnetic energy available, in terms of effective radiated power from an RF source (or antenna), varies widely according to source type, as shown in Table 2. The visible-light example (light bulb) is provided for comparison, but its energy output is primarily in the infrared and visible portion of the electromagnetic spectrum. Among RF sources, cellular telephone base stations are at the low end when considering the strength of the source of RF power.

Radiofrequency exposure is typically quantified as RF energy flux per unit area, for example, watts of RF energy crossing a square meter of area (*W/m*^2^). Alternatively, the intensity of radiowaves can be given in terms of electric field intensity, where the units are volts per meter (*V/m*). These two metrics are mathematically related to each other when considering locations many wavelengths distant from the antenna (or the RF source). That is, the energy flux per unit area (*S*) is proportional to the square of the electric field intensity (*E*):


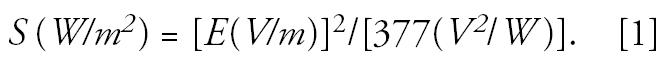


For example, RF energy of 1 W/m^2^ is equal to 19.4 V/m, and 10 W/m^2^ is equal to 61.4 V/m (because of the squared dependence between *S* and *E*).

The relevant RF energy flux (in terms of potential health impacts) is at exposure points where people may intercept the RF energy, and is measured in power per square meter of surface area. A comparison of energy fluxes in this regard is given in Table 3, which compares both RF and non-RF sources. It can be seen that more energetic electromagnetic waves (visible light, infrared waves) are normally present at energy flux levels more intense than the maximum allowable RF intensities in the cell telephone band. In fact, our body surfaces radiate sufficient infrared energy that they are easily seen by “night vision” cameras. Because of their warm temperature, our bodies also emit RF energy in the microwave band (~ 30–300 GHz) at about 0.003 W/m^2^.

Table 3 also illustrates that the amount of electromagnetic energy that is present due to cellular telephones and cellular base stations is quite small in comparison to both electromagnetic energy sources generally and RF sources in particular.

Within the home and office environment, a variety of other sources of RF energy are used. Table 4 lists frequency ranges and maximum output powers from typical device classes in home and office environments (Kühn et al. 2006). The peak output power represents the maximum peak output of the investigated device classes. The International Commission on Non-Ionizing Radiation Protection (ICNIRP 1998) identified allowable public exposure levels for electric field (E-field) over these frequencies ranging from approximately 30 V/m to 60 V/m.

Spot measurement data often show that, where a particular RF source is the focus of concern, other, less visually obvious sources may give greater contributions to exposure. The data also show that exposures vary greatly, even at similar distances from base stations, illustrating the dramatic effect of the local environment on RF signals through physical processes such as reflection, diffraction, and mutual interference of signal elements traveling through multiple, different paths (Ardoino et al. 2004; Mann et al. 2006).

For assessing occupation RF exposure in the context of base station antennas, a “compliance boundary” can be used, defined so that for personnel outside the boundary, RF levels are low enough to be in compliance with relevant safety standards. The size and shape of a given compliance boundary varies with frequency, with type of antenna, and with antenna power output. For a typical base-station antenna running at 25 W, the compliance boundary has the shape of a cylinder with a diameter of 3 m, and a height corresponding to the antenna height plus about 0.5 m. The height is centered on the antenna, and the cylinder wall begins about 0.1 m behind the radiative front of the antenna and extends to about 2.9 m in front of the antenna. This virtual space encloses the volume where the RF signal may be in excess of ICNIRP occupational standards, but for all distances outside this cylinder, RF levels are low enough to be considered safe (Mild et al. 2006). By comparison, in occupations such as “plastic sealers,” RF levels can be considerably higher than in the close vicinity of base antennas (Wilen et al. 2004).

Both measurement surveys and theoretical predictions show that RF levels from base stations and wireless technologies generally decrease with distance from the device (with focused antenna arrays, maximum ground level RF is 50–300 m from the antenna base). That is, the greater the distance from the antenna, the lower the RF. Under conditions typical for public exposure to base stations and for wireless consumer devices, the RF energy fluxes are > 100-fold below international RF guidelines for public locations. However, in very close proximity to base-station antenna elements under occupational conditions (i.e., when performing maintenance on an operating antenna), or immediately adjacent to wireless local area network (LAN) and Bluetooth transmitters, there is the possibility that RF absorption limits for the general public may be exceeded (Kühn et al. 2006). Thus, there is a need to ensure that, under normal operating conditions, these devices comply with the international limits. In the case of nonoccupational exposure to RF from base stations, the most common circumstance is that the contribution of base stations to a person’s total RF exposure is minimal. (“Bluetooth” is a term generally designating digital wireless communication among personal-computer-associated devices—i.e., “digital enhanced cordless telecommunication” between laptops, personal computers, personal digital assistants, cell phones, printers, digital cameras, etc. The name “Bluetooth” refers to the 10th-century king of Denmark, King Harold Bluetooth, whose diplomacy led warring parties to negotiate with each other. The inventors of the Bluetooth technology thought this a fitting name for a technology that allowed different devices to talk to each other.)

## Mechanisms for RF Effects, and Role of RF Modulation

Cellular telephone radio waves transmit information that is encoded into electromagnetic waves by means of “modulation,” which refers to the patterns of change in the frequency and/or amplitude of the RF carrier wave. As cellular telephone technology has advanced, the modulation patterns have become increasingly complex, and the question arises as to whether a high-frequency modulated RF wave might have greater potential for health effects than a pure sinusoidal RF wave. The applicability of fundamental physics to all systems, and particularly to biology, permits one to conclude that modulation is unlikely to lead to unexpected RF interactions with ions, molecules, cells, and organisms—i.e., interactions substantially different from unmodulated RF (Valberg 2006).

Modulation introduces a spread of frequencies into the RF signal, but the frequency bandwidth of the net RF signal generally remains a small fraction of the central, carrier frequency. This means that the most representative frequency range for modulated electromagnetic waves is that of the (high-frequency) RF carrier, not the (low-frequency) modulation pattern. Even though the power of the RF signal may vary in step with the modulation frequency, the transmitted RF spectrum contains no electromagnetic waves at the modulation frequency. Characteristics of the bandwidth, carrier wave, and modulation depth for some typical RF sources are summarized in Table 5.

As Table 5 illustrates, parameters of potential biological significance include the frequency content of the signal (ratio of modulation frequency to carrier wave frequency), the ratio of peak-to-average RF wave amplitude, the central frequency of the RF (carrier wave), and the modulation frequency (typically ~ 0–10 kHz).

Tumorigenicity studies in laboratory animals provide some insight as to the biological effects of RF modulation and of RF exposure generally. Elder recently reviewed 36 publications that reported tumorigenicity assays in rodent species, after RF exposure in the frequency range applicable to mobile telephony {Elder JA, personal communication; [these studies are in the WHO EMF Database (WHO 2006a)]}. Table 6 summarizes the results of Elder’s compilation of the animal tumorigenicity literature, grouped by type of RF modulation and with each result at a different frequency, different modulation, or different power level counted as a separate “test,” resulting in 68 separate tests in the 36 publications. The species were primarily mouse and rat, and the RF frequencies ranged from 435 MHz to 9,400 MHz. Table 6 also lists the modulation types tested by the investigators.

Table 6 reveals a preponderance of null results, and Elder observed that the more recent, better-designed studies were overall negative. For the seven positive outcomes, the authors of the studies were not able to conclude that any given positive result fully met criteria of validity such as dose response, consistency, and reproducibility. It should be noted that, when testing at a *p* < 0.05 significance level, for 68 tests, about three to four positive results would be expected by chance alone. These data do not tend to support the idea that modulated RF is more potent than non-modulated RF, because three results reaching *p* < 0.05 appeared in 15 tests of nonmodulated RF (CW only), i.e., 20%, whereas the remaining 4 results reaching *p* < 0.05 appeared in 63 tests of modulated RF, i.e., 6%. Overall, the weight of evidence in animals exposed for extended periods, up to lifetime exposures of 2 years, at a variety of frequencies and modulations, suggests that exposure to modulated RF does not increase risk of tumor development (Elder JA, personal communication; Dasenbrock 2005).

Mechanistic considerations are central to examination of the role of modulation in biological effects of RF because living organisms rely on the same physical laws that govern all systems (Durney and Christensen 2000). Physics forms the basis of chemistry, which forms the basis of biology, which forms the basis of medicine. Hence, even though moving up this progression is marked by an increase in complexity, each successive layer must obey the fundamental laws found to be valid for the layer below. The most fundamental level rests on the laws of physics, which have been exhaustively validated by experimentation and through internal consistency. The principles behind radiofrequency waves—namely, Maxwell’s laws of electromagnetism—are accepted to be invariant in time and space, and their accuracy in describing the interactions between electromagnetic fields and matter underlies the functioning of virtually all technology. No exceptions have been found despite constant challenges. Likewise, physics has been found to be valid in complex systems, encompassing chemistry, biology, technology, and medicine (Polk and Postow 1996). Simple conservation laws (e.g., energy, motion, charge, momentum) are universally applicable, and biology is no exception.

Any biological interaction mechanisms capable of detecting the difference between a modulated RF signal and an nonmodulated RF signal must be either be *a*) fast enough to respond to and detect changes in the central RF frequency, or *b*) sensitive to the RF power changes occurring at the modulation frequency. For *a*), scientists have not been able to identify biological structures capable of the necessary high-frequency RF tuning or bandwidth discrimination. For *b*), being sensitive to the power changes in the signal would require a biological structure that is nonlinear at low power levels (i.e., that can “rectify” the RF), which has not as yet been identified by biologists or anatomists. If “rectification” by biological structures were to occur, it remains difficult to envision how a tiny amount of modulated RF power, occurring at “nonthermal” levels below existing exposure RF standards, would lead to deleterious effects on biological systems. Living systems have considerable thermal output, overall thermal inertia, and efficient thermal regulation. Those nonthermal mechanisms that rely on nonlinear responses (e.g., breakdown of the cell membrane) have high RF–electric-field thresholds. RF levels capable of electroporation would in themselves produce hazardous tissue heating (Adair et al. 1997).

For RF energy to change physiological function, initiate dysfunction, or cause the onset of disease in humans or animals there must exist a mechanism by which the physical forces exerted by electric and magnetic fields on charged particles alter molecules, chemical reactions, cell membranes, or biological structures (Parkinson 1985). RF is a physical not a chemical agent, and the biological plausibility of initiating a process that leads to adverse health effects must be assessed with this in mind. The initial physical step is illustrated in the following causal chain by which RF interaction effects could occur:


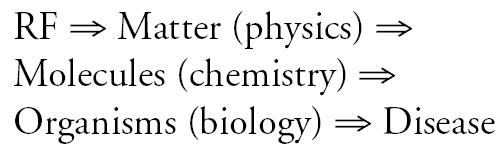


**Figure 1:**
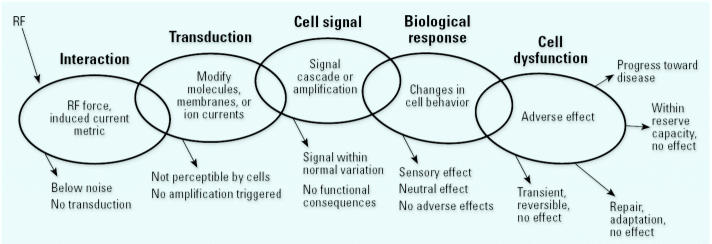
The causal chain leading from an exposure to disease has multiple steps, each of which may or may not trigger the next step. For RF interactions with molecules, cell structures, or tissues, the transduction mechanism is a crucial first link in the causal chain. By definition, the electric and magnetic fields in RF waves can exert force on electrically charged particles.

This process is illustrated in more detail in Figure 1. Biological processes in living organisms include many interactions among electric charges (on ions, molecules, proteins, and membranes). Hence, it is clearly possible that exposure to RF, the electromagnetic fields of which can exert forces on fixed and moving charges, might have the potential to modulate biological function. For RF to cause or exacerbate disease in humans, the RF electric fields would have to trigger an initial transduction step, and then also begin a cascade of sequential steps that leads to a disease outcome. As Figure 1 illustrates, the causal chain would begin with human exposure to RF. To complete the first step, RF would interact with biological molecules (or structures) in such a way as to alter their size, shape, charge, chemical state, or energy. In this initial “transduction” step, some absorption of RF energy must occur or there can be no effect.

Figure 1 shows that, for observable biological (and possibly health adverse) effects to follow transduction, sequential events at the molecular, cellular, and tissue level are required. Each step can lead to a variety of possible end points, and only certain outcomes lead farther down the causal chain. The outcome “Progress toward disease” in the upper right corner (Figure 1) is only one of many possible outcomes, and it requires specific triggering of intermediate steps. There are multiple points in the causal chain where the signal produced by a weak preceding step might be within normal variations and, therefore, may well have no further functional consequences beyond this point in the causal chain.

For low levels of RF (nonthermal levels) the first, mechanistic or transduction step in the multistep pathway has eluded intensive scientific search (Veyret 2006). Even the well-known “hearing effect” for pulsed high-level microwaves (Aran et al. 2004; Elder and Chou 2003) is based on small thermal fluctuations, even though the time-averaged thermal input is nil. Because the health and viability of the human body depends in a fundamental way on the normal structure and function of large molecules (e.g., proteins, nucleic acids, carbohydrates, and lipids), any proposed RF mechanism must predict how RF could interfere with or modify the normal synthesis, function, or degradation of these molecules. The RF interaction mechanism(s) would then predict thresholds of exposure effectiveness in terms amplitudes, frequencies, time of onset, intermittency of exposure, homogeneity/heterogeneity of amplitudes and frequencies, exposure duration, transients, polarization, and so on. Moreover, mechanistic understanding would address the possibility that children may react to RF more effectively than adults. However, our current understanding does not bear out this hypothesis (Christ and Kuster 2005).

The magnitudes of endogenous forces that are known to act at the cellular level to modify protein structures have been measured and can serve as a basis for comparison to forces produced by RF fields (Titushkin and Cho 2006; Valberg 2006). Thus, the mechanistic constraints on having observable, force-generated effects on biological systems can be appreciated by ranking electric field forces from RF signals relative to forces normally generated (or sensed) by molecules in living systems. Using the electric field strength in tissue corresponding to a specific absorption rate (SAR) of 2 W/kg, which is the maximum level allowed for cell telephones (averaged over 10 g of tissue), we can estimate the associated internal electric field to be ~ 45 V/m at 1 GHz. In evaluating possible bioeffects of this electric field, a useful unit of force is the piconewton, which is one-trillionth (10^−12^) of a newton (N) of force. The weight of 1 cm^3^ of water (mass = 1 g) is ~ 0.01 N. The mass of a human cell, approximately 10 μm in size would be 10^−9^ g, and would weigh (in air) approximately 10^−11^ N, or 10 pN. Considering a protein molecule that has 100 unbalanced electric charges (+ or −), we can calculate the maximum force of 45 V/m on this molecule to be ~ 0.0007 piconewtons (pN). This is > 10,000-fold smaller than the smallest forces known to modify protein molecule function. For example, the electric force on a 100-fold-charged protein molecule embedded in the cell membrane would be about 160 pN, because of the electric field from the normal resting cell-membrane potential (~ 70 mV over 7 nm). That is, typical cell membrane voltages result in robust electric-field forces on cell-membrane proteins, which are known to modify function, such as open and close ion channels. Forces 10,000 times smaller can be expected to be without effect.

Examination of the magnitude of the possible biophysical interactions (thermal, photon, force) of electromagnetic fields with living matter shows that, under modulated RF exposure conditions allowed by the current safety limits, there does not appear to be an overlooked hazard specific to RF modulation, with the possible exception of RF in the form of very short, high-intensity pulses, which are far more energetic than any pulses encountered in cellular telephone technology (Adair 2003; Challis 2005; Foster and Repacholi 2004; National Council for Radiation Protection 2003). For RF levels below the established standards (modulated or not), scientific research has not identified reproducible and plausible mechanisms by which biological effects can be caused in living systems. Because of the safety factors built into guideline levels, even RF somewhat exceeding permissible levels would yield amounts of thermal energy absorbed that are within the adaptive capacity of the body, and would not be likely to lead to disease (Ebert et al. 2005).

Consideration of the possible mechanisms by which mobile-telephony RF can interact with the body does not reveal a means by which modulated RF, specifically, could lead to adverse effects.

## Lines of Evidence on Possible RF Health Effects

### Cancer

Most cellular studies indicate an absence of effects on DNA, and generally biological data, including results from animal studies, do not demonstrate an increased risk of cancer from exposure to RF fields (Vijayalaxmi and Obe 2004). Reassuringly, with respect to base station RF fields per se, the levels of exposure are very low compared with those at which biological effects of any kind have been observed. However, because some biological experiments continue to suggest new possibilities for RF interactions, more scientific research is called for. It has been pointed out that, if one were proposing new chemicals for commerce or new pharmaceuticals, the quality of most available RF studies would not be acceptable for registration with the responsible authorities (Dasenbrock 2005).

The ongoing INTERPHONE collaboration is a multicenter, comprehensive study on mobile phones and cancer. It is coordinated by the International Agency on Research on Cancer (IARC), a specialized cancer agency of the WHO, and researchers in 13 countries are taking part using a common protocol. The INTERPHONE protocol is a population-based, case–control study correlating head and neck tumors with mobile-phone use by persons 30–59 years of age who reside in the study regions. Exposure assessment is reliable because it is based on individual records of cell phone use. Because of pooling of data from all participating centers, the study is statistically powerful.

For example, the risk of acoustic neuroma in relation to mobile phone use has been assessed via six population-based, shared-protocol, case–control studies in four Nordic countries and the United Kingdom. The authors concluded that there was no association of risk with duration of use, lifetime cumulative hours of use, or number of calls, for phone use overall or for analogue or digital phones separately (Schoemaker et al. 2005). Recent results from INTERPHONE have reported lack of brain tumor or acoustic neuroma risk in Japan (Takebayashi et al. 2006), Germany (Berg et al. 2006; Schuz et al. 2006), and in a meta-analysis of five Northern European countries (Lahkola et al. 2007).

There have also been other studies of mobile telephone users, particularly on brain tumors (and less often on other cancers and on symptoms). Results of these studies to date give no consistent or convincing evidence of a causal relation between RF exposure and any adverse health effect (Ahlbom 2006; Ahlbom et al. 2004; Lonn et al. 2005). A 4-year British survey released in 2006 showed no link between regular, long-term use of cell phones and the most common type of brain tumor, glioma (Hepworth et al. 2006). A German study did find an elevated risk of glioma in long-term users, but the increase was not statistically significant. The authors concluded that no overall increased risk of glioma or meningioma was observed among these cellular phone users (Schuz et al. 2006).

Another approach that has been used is to compare temporal trends in disease rates with temporal trends in prevalence of cell phone use. For example, trends in acoustic neuroma incidence in England and Wales were found not to lag behind trends in cell phone use in a correlated fashion (Nelson et al. 2006). Because increases in acoustic neuroma incidence predate or parallel rates of cell phone use, they likely reflect changes in reporting and diagnosis, and the temporal trends go counter to what would be expected if RF exposure played a role.

Several epidemiologic studies of potential cancer risk have used proximity to commercial broadcast transmission towers as the measure of RF exposure. However, individual RF exposures are not necessarily related to distance. None of these epidemiologic studies has provided sound evidence that RF exposure from the transmitters increased the risk of cancer or any other health effect [see summary by Jauchem (2003)]. The reporting of cancer “clusters” around RF broadcast transmitters and mobile phone base stations has heightened concern among the general public, but given the random nature of the distribution of cancers in the population it is not surprising statistically that such clusters should appear. Also, given the ubiquity of base stations, one would expect that a base station being near existing cancer clusters is a likely occurrence. Hence, reliable scientific evidence on how the distribution of cancer and other diseases in the population might be related to environmental factors (e.g., cellular telephone RF exposures) can best be obtained through carefully planned and executed epidemiologic studies such as INTERPHONE.

### Noncancer health effects

Some changes have been reported in relation to cardiovascular function, but these findings have been in operators of broadcasting stations (Vangelova et al. 2006). From the weight of evidence and the very low exposure levels associated with base stations, there is no clear evidence of any adverse effect from such exposures (Feychting 2005; Feychting et al. 2005).

### Potential neurologic and behavioral effects

Laboratory studies with volunteers have investigated whether low-level exposure to RF fields associated with mobile phones can affect brain function and behavior. Reported reactions to assumed RF exposure include a wide variety of nonspecific symptoms. Most commonly reported symptoms are sleeplessness, fatigue, dizziness, digestive disturbances, and concentration difficulties. By and large, well-controlled and -conducted double-blind studies have shown that symptoms are not correlated with RF exposure. There are also some indications that these symptoms may be caused by preexisting conditions such as stress reactions resulting from worrying about perceived RF health effects rather than the RF exposure per se. To date, only subtle and transient effects have been reported, and any implications for health remain unclear and unlikely (Cosquer et al. 2005). Exposures used in these studies are similar to those to the head from mobile phone use, rather than to the much lower RF levels associated with general public exposure from base stations.

Reviews of the evidence on electromagnetic hypersensitivity have been conducted (Fox 2006). An extensive systematic search identified relevant blind or double-blind provocation studies of individuals potentially hypersensitive to the presence of EMF. A meta-analysis found no evidence of an improved ability to detect EMF in “hypersensitive” participants. That is, it was concluded that weak electromagnetic fields are not likely to be causative factors for neurological symptoms (Rubin et al. 2005, 2006a, 2006b). An investigation into possible differences in blood cells between patients reporting EMF hypersensitivity and normal patients did not find any differences in lymphocyte response to RF from GSM mobile telephones (Markova et al. 2005). Other investigators have likewise concluded that “based on the limited studies available, there is no valid evidence for an association between impaired well-being and exposure to mobile phone radiation” (Seitz et al. 2005).

However, it is important to recognize the plight of people suffering from “hypersensitivity reactions.” The WHO recently issued a fact sheet about people reporting nonspecific symptoms that they relate to RF fields from base stations and other EMF devices. Details can be found at WHO (2005). Moreover, E. Fox in the United Kingdom is continuing to analyze possible electromagnetic hypersensitivity reactions, and a report on the findings has been published (Eltiti et al. 2007).

### Summary on RF health effects

The accumulated evidence does not establish the existence of adverse short- or long-term health effects from the signals produced by base station and local wireless networks. In fact, for similar RF exposure intensities (watts per square meter), the body absorbs about 5 times more of the RF energy from FM radio and television frequencies (around 100 MHz) than from base station frequencies (around 1–2 GHz). It is reassuring to note that radio and TV broadcast stations have been in operation for > 50 years, and health statistics have not demonstrated adverse health consequences.

### Development of national and international RF guidelines

The heath-effect guidelines of ICNIRP in the mobile telephony frequency spectrum range from about 40–60 V/m (4.3–10 W/m^2^) (ICNIRP 1998). The ICNIRP guidelines have been widely accepted (> 30 countries worldwide) and, for example, are consistent with Health Canada (1999), U.S. [American National Standards Institute/Institute of Electrical and Electronics Engineers (2006), Federal Communications Commission (2006)], UK [National Radiation Protection Board/Health Protection Agency (NRPB/HPA 2004a, 2004b)], and Australian [Australian Radiation Protection and Nuclear Safety Agency (ARPANSA 2002)] standards. However, some countries and regions have adopted more stringent guidelines without specifically justifying them on the basis of available scientific evidence. In contrast to the ICNIRP levels, the following are some examples of these more restrictive guidelines, in the mobile telephone frequency range (Baumann 2006; Vecchia 2006):

- ICNIRP Guidelines: 40–60 V/m or 4.3–10 W/m^2^
- “Italian Exposure Limit”: 6 V/m
- “Paris Charter”: 2 V/m, 24-hr average, indoors
- “Salzburg Protection Value”: 1 W/m^2^
- “Swiss Regulation”: 4–5 V/m at full power.

The issues that most often drive more localized RF guidelines are not established health risk per se, but rather risk perceptions (Siegrist et al. 2005). In this regard, the “Precautionary Principle” is often invoked—“better safe than sorry”—part of which involves taking “protective measures without having to wait until the reality or seriousness of those risks becomes apparent.” One expression of how “protective measures” might be applied to RF levels is:

The proposed [RF] standard also recommends that it is generally sensible to minimize exposure which is unnecessary or incidental to achievement of service objectives or process requirements, provided this does not introduce other risks and it can be readily achieved at modest expense. (ARPANSA 2001)

The term “modest expense” implies some type of cost–benefit analysis. Appropriate application of the Precautionary Principle requires that the policies be tailored such that the time, effort, expense, and risk of any “protective measures” be commensurate with what the society expends on other public risks of similar magnitude. However, if scientific research is not able to establish “apparent risks” in a quantitative way, making such a calculation is problematic.

If a basis for precautionary limits cannot be provided, then the danger behind promoting arbitrary limits and superfluous safety factors is that reliance on logical, science-based policy will be undermined in favor of unreasoned, fear-based, poorly thought out actions. Such actions, rather than providing reassurance, will likely trigger concerns, amplify unwarranted anxieties, and likely divert scarce resources into areas yielding little or no public health benefit (Barnett et al. 2006; Wiedemann and Schutz 2005). Despite unavoidable uncertainty and other limitations of the scientific method, science remains our best source of knowledge about how the world works and how we can rely on natural laws to understand interactions between the environment and living things.

Despite reassuring scientific evidence, some people perceive risks from mobile telephony RF exposure as likely and possibly severe. Several reasons for public fear have been proposed, including media announcements of new and unconfirmed scientific studies, leading to a feeling of uncertainty and a perception that there may be unknown or undiscovered hazards. Risk perception cannot be understood as a monolithic concept; rather, communicating “risk” is a unique challenge because it focuses on three major elements that are difficult to convey to a lay public: complexity, uncertainty, and ambiguity (Renn 2004, 2006).

The first barrier to easy risk communication is complexity. In everyday life, people perceive causal connections to be simple. In most scientific assessments of risk, causal connections are highly complex. Causes and effects are not obvious, and there are many intervening variables that obscure possible relationships. Often facility with quantitative methods is required, and appreciation of the fact that “the devil is in the details.” Even though most scientists agree that health impacts of mobile telephony RF are unlikely (but not impossible), a lay audience will often assume that complexity is used to “pull the wool over their eyes.”

The second barrier to explaining risk is uncertainty. Most risks have a hypothetical component, and we cannot say with certainty that *x* exposure will cause (has caused) adverse outcome *y*. For example, science can rarely make definitive statements about what has “caused” a particular disease like cancer. The best we can do is to calculate probabilities linked to different causal pathways. In terms of risk communication, conveying the different types of uncertainty to a lay audience poses major challenges. People misunderstand uncertainties as being an indicator for bad science or sloppy risk assessment. In the face of uncertainty, a lay audience will typically resort to the simplistic strategy: “Better safe than sorry.”

The third component of risk that is difficult to communicate is ambiguity. Ambiguity refers to the existence of divergent or contested interpretations and perspectives on the severity associated with a potential health threat. Not only can scientific interpretations have an ambiguous component, but ambiguity also emerges with regard to selection of appropriate values, priorities, assumptions, ethics, distributions of risk, and quality of life parameters to be applied. In the face of “duelling experts,” a lay audience will often assume that the correct interpretation is midway between the two perspectives offered, because a lay audience has limited ability to recognize fringe positions.

To deal with these problems in risk communication, the public needs to be provided access to accurate information and education on scientific consensus positions. First, the solution to complexity is not a simple prohibition of a given technology. Rather, it must be recognized that scientific inquiry can test for harmful effects, but it can never prove that something is safe. This asymmetrical relationship is difficult to communicate, but one approach is to encourage people to realize that absolute safety is not a requirement that they can or do impose on any societal activity (e.g., public transportation, food supply, medical procedures, prescription drugs). Second, science deals with uncertainty by incorporating safety factors. That is, safety guidelines are not bright boundary lines between “good” and “bad,” but rather they incorporate an adequate margin of safety so that even exceeding the exposure guideline would not lead one to anticipate ill effects. Third, in dealing with ambiguity, lay audiences need to be educated that the scientific consensus does not necessarily lie midway between the opinions they are offered. Hypothetical risks need to be recognized at such, and in a scientific, secular world, explanation patterns that are counter-factual, paranormal, and metaphysical are not valid. Although the natural human ability to sympathize with the victims of disease is to be admired, the associated desire to pin the blame for inexplicable diseases on a nearby environmental factor is misguided. That is, when individuals contract diseases, and when we have little idea as to what specifically caused the disease in question, that ignorance somehow sounds like evidence in favor of focusing on “unknown” risks such as RF or EMF. But this lack of knowledge is not evidence.

Perhaps the most important element of risk communication is to assure audiences that RF standards have been and continue to be under ongoing scrutiny. Large numbers of scientists, medical doctors, and public health professionals of disparate orientations and areas of expertise sift existing data and contribute new data in an ongoing risk assessment effort. The vast majority of human cancers are likely caused by unavoidable environmental exposures (e.g., viruses, diet, lifestyle, sunlight, background ionizing radiation) or to processes inherent to life itself (e.g., genetic instability, copying errors in DNA, endogenous hormones, creation of mutagens and free radical molecules by metabolism of food, production of reactive chemicals for microbicidal defense) (Gotay 2005; Henderson et al. 1991; McKean-Cowdin et al. 2000; Wogan et al. 2004). In scientific risk assessment, one compares the ability of the exposure of interest to increase risk above these baseline, natural processes.

## Summary Potential for Health Effects from Wireless Technologies

As outlined above, our best scientific understanding indicates that there are no health consequences of base-station RF exposure, and no adverse effects are foreseen at the RF levels typical of cellular telephone technology. This viewpoint is not only consistent with the conclusions of the WHO workshop on “Base Stations and Wireless Networks,” but it is also consistent with numerous other public health reviews on the safety of wireless technologies, although most public health agencies continue to favor “additional research.” Some of these blue-ribbon, consensus-group conclusions are mentioned below.

ICNIRP (1998) has developed guidelines to protect human health from exposure to EMF across the RF spectrum. These ICNIRP guidelines have been adopted by > 30 countries. Certain countries have instituted standards limiting emissions from cellular telephone base stations that are significantly below recommended ICNIRP limits. Such additional restrictions are not based on any known health effects, but rather tend to be either a precautionary measure or an “as low as reasonably achievable” (ALARA) measure that requires base station transmissions to be no more than required for providing a good service.

Several groups in Great Britain have evaluated potential health effects of RF. The Advisory Group on Non-Ionizing Radiation (2003) updated the year 2000 report of the Independent Expert Group on Mobile Phones (2000) and concluded that “exposures due to living near to base stations are extremely low, and the overall evidence indicates that they are unlikely to pose a risk to health.” (Advisory Group on Non-Ionizing Radiation 2003).

The UK Health Protection Agency (formerly the National Radiation Protection Board) (NRPB/HPA 2004a) also has concluded that RF energy can potentially cause health effects only if people are exposed to RF levels significantly exceeding international limits. That is, they recommended that exposure to EMFs (0–300 GHz) in the UK be based on the 1998 guidelines issued by ICNIRP (NRPB/HPA 2004a).

In a specific review of cellular telephone technology (NRPB/HPA 2004b), the agency proposed that even though “there is a lack of hard information showing that the mobile phone systems in use are damaging to health,” they continued to endorse a “precautionary approach” to the use of mobile phone technologies.

The Health Council of the Netherlands (Health Council 2002) has prepared a report on the potential risks of EM fields from mobile telephones. The report concluded that “the EM field of a mobile telephone does not constitute a health hazard, according to the present state of scientific knowledge.” Moreover, the review committee concluded that “the scientific information concerning non-thermal effects discussed in this report provides no reason to apply the precautionary principle and lower the SAR limits for partial body exposure” (Health Council 2002). A 2005 Health Council update concluded that “the Committee therefore disagrees … that a connection has been found between living in the proximity of a base station and the occurrence of cancer” (Health Council 2005).

ARPANSA prepared a fact sheet titled “What about base stations and telecommunication towers–are there any health effects?” ARPANSA concluded that “the weight of national and international scientific opinion is that there is no substantiated evidence that RF emissions associated with living near a mobile phone base station or telecommunications tower poses a health risk” (ARPANSA 2003a). ARPANSA also evaluated the potential for risk to children and concluded that “the balance of evidence does not indicate a risk to the health of people, including children, living in the vicinity of base stations where the exposure levels are only small fractions of the ARPANSA Standard” (ARPANSA 2003b).

The Royal Society of Canada has an “Expert Panel on Potential Health Risks of Radiofrequency Fields from Wireless Telecommunication Devices,” and their most recent update (2004) notes that “all of the authoritative reviews completed within the last two years have concluded that there is no clear evidence of adverse health effects associated with RF fields” (Royal Society of Canada 2004).

The advice of the U.S. Health Physics Society (a professional society of specialists in radiation safety) is that there is no reason to believe that cellular base station towers could constitute a potential health hazard to nearby residents or students (Health Physics Society 2006).

At present, the only established effects that can result from excessive exposure to RF energy are related to tissue heating. Although RF energy can be absorbed by living organisms to some degree at any frequency, available data do not demonstrate adverse health consequences at exposure levels below internationally accepted limits, which do not allow significant heating. In summary, none of the recent research or reviews of research have concluded that permissible RF exposure levels from mobile phones and their base stations lead to adverse health consequences.

Although scientists generally assign low priority to conducting research on base stations or other wireless technologies having such weak RF signals, some gaps in knowledge still exist (Repacholi 1998). Research recommended to fill these gaps can be found in the WHO RF research agenda (WHO 2006b).

## Figures and Tables

**Table 1:** Typical RF sources contributing to modern-day radio-wave background.

RF Source	Frequency (MHz)	Exposure potential
AM commercial radio	0.5–1.7	U+
Ionosphere research programs (e.g., HAARP)	2.8–10	L
FM commercial radio	88–108	U+
VHF commercial television (analog)^a^	54–88, 174–216	U+
UHF commercial television (analog and digital)	512–700	U+
Maritime mobile, radiolocation, radio-navigation (e.g., LORAN)	0.003–0.30	L
Radar (aviation, marine, police)	10,000–33,000	L
Millimeter-wavelength radar (meteorological, military)	~ 100,000	L
Satellite transmissions (global positioning, military)	220–400	U
Satellite transmissions (television)	4,000–6,000	U
Amateur (ham) radio operators, international short-wave broadcasts	~ 50	U
Cellular telephones, analog	806–890	U
Cellular telephones, GSM (Asia, Europe)	890–960	U
Cellular telephones, digital	1,850–1,990	U
Dispatch radio: (pagers, aviation, marine, fire, emergency, police)	900–950	U
Fixed microwave links (computers, television, telephone, military)	~ > 30,000	L
Cordless telephones, baby monitors, wireless toys, wireless telemetry	27–60, 900, 2,400, 5,800	L
Computer monitors, wireless computer connectivity, RF identification tags (e.g., Bluetooth, WiFi)	~ 1,900, ~ 2,500, ~ 5,700	L
Remote controls, light dimmer controls, door-openers, surveillance devices	Broadband	L
Microwave ovens, diathermy machines	2,450	L+
Industrial scientific and medical (ISM) band data links	~ 2,400, ~ 5,400	L
RF noise (lightning, solar flares, fluorescent fixtures, neon lights, spark ignition, power-line corona discharge)	Broadband	U

Abbreviations: +, those sources, among the ones listed, that typically contribute to the major fraction of total ambient RF exposure; GSM, global system for mobile communications; HAARP, high-frequency active auroral research program; LORAN, long-range radio navigation; L, localized RF sources; U, ubiquitous RF sources.

a The VHF band is split into two parts, with FM radio in the middle.

**Table 2:** Approximate radiated-power emission strength for sources of electromagnetic waves.

Source	Energy (W)
Cellular telephone handset	~ 0.6
Single light bulb (visible and infrared waves)	100
Single ham radio antenna	1,000
Array of cellular phone base station antennas	1,200
Typical AM radio station transmitter	50,000
Typical FM radio station transmitter	100,000
Typical UHF TV transmitter	1,000,000

**Table 3:** Incident energy from a broad spectrum of sources of electromagnetic energy.

Source	Energy flux (W/m^2^)	Electric field (V/m)
Sunlight at noon^a^	1,370	
1 m from a 1,500-W electrical heater unit^b^	480	
On black body surface at 37°C (λ_max_ ~ 10 μm)^c^	520	
Microwave oven, RF leakage standard	50	140
1 m from a 100-W light bulb^d^	8	
Cell telephone (2 GHz) public guideline^e^	10	61
Cell telephone (850 MHz) public guideline^e^	4.3	40
RF levels near cellular base antenna (calculated)	0.05	4.3
Average urban RF levels, TV and radio^f^		0.4–0.7
Average urban RF levels, cellular telephony^f^		0.1–0.3

a The average amount of solar energy reaching the earth’s atmosphere is defined as the solar constant = 1,370 W/m^2^.

b Assuming that a reflector behind a 1-m-long heating element directs the 1,500 W of energy into the half-cylinder in front of the heater, the surface area at 1-m radius is 3.14 m^2^, so 1,500 W divided by 3.14 m^2^ is 477 W/m^2^.

c Wien’s Law states that the wavelength, λ, at which most power is radiated by a body at temperature T is λ = 2898/T = λ (μm), where T is degrees Kelvin and the wavelength is given in micrometers. The Stefan-Boltzmann Law states that the energy flux from a black body at temperature T is given approximately by Φ, where Φ = σ T^4^ W/m^2^, where σ is the Stefan-Boltzmann constant (5.67 × 10^−8^ W/[m^2^K^4^]).

d Assume spherical radiation, at 1 m, the surface area is 4π*r*^2^ = 12.6 m^2^. Hence, 100 W/12.6 m^2^ ≅ 8 W/m^2^.

e ICNIRP reference level for general public exposure (ICNIRP 1998).

f Anglesio et al. (2001).

**Table 4:** Sources and levels for indoor RF-communications technologies.

Technology	RF range (MHz)	Peak output power (mW)	Max E-field at 20 cm (V/m)
Digital enhanced cordless telecommunications	1,880–1,900	250	11.5
Wireless peripherals interconnection (Bluetooth)	2,402–2,480	100	3.1
Wireless LAN (IEEE 802.11b/g)	2,400–2,484	100	3.9
Wireless LAN (IEEE 802.11a/h)	5,250–5,350, 5,470–5,725	200	3.9
Wireless personal computer peripherals	27–2,400	10	< 1.5
Baby surveillance devices	27–2,400	500	8.5
Cellular telephone base station RF in proximity of residences^a^	900–1,800	—	0.1–1.0

Abbreviations: IEEE, Institute of Electrical and Electronic Engineers, Inc.; Max, maximum.

a Typical E-field levels in proximity to cellular telephone base stations (< 200 m) (Coray et al. 2002).

**Table 5:** Modulation characteristics of RF fields in different applications.

Technology	Typical modulation	Ratio, BW/CW frequency	Peak/avgerage amplitude	Examples [CW frequency (GHz)]
AM broadcasting	Amplitude	Very small << 1	~ 2	AM radio (~ 0.001)
FM radio and television	Frequency	Very small << 1	~ 1	FM radio (~ 0.1)
Mobile communications	Pulse and frequency	Very small << 1	~ 10	UMTS, TETRA, GSM, TDMA, CDMA, (~ 0.4–2)
Radar	Pulse	Modest < 1	100	Airport radar (~ 4)
Ultra-wideband, spread spectrum	Short pulse	Large ~ 1	100	Military applications (~ 2–20)

Abbreviations: BW, bandwidth; CW, carrier wave. Adapted from Foster and Repacholi (2004).

**Table 6:** Modulation schemes tested for tumorigenicity in animal models {Elder, personal communication [all the studies are in the WHO EMF Database (WHO 2006a)]}.

			Effect on tumor incidence	
MHz, central frequency	Type of modulation tested	No. of tests made	Increase	No increase
800–9,400	CW	15	3	12
915	AM	5	0	5
836–903	FM	4	0	4
435, 2,450	PW	3	1	2
848, 1,763	CDMA	5	0	5
849	DAMPS	1	0	1
836	FDMA	1	0	1
900–902	GSM	22	3	19
836–1,500	TDMA	9	0	9
1,616	Iridium	2	0	2
5,680	UWB	1	0	1
Total no. of tests		68	7	61

Abbreviations: AM, amplitude modulated; CDMA, code division multiple access; CW, carrier wave (unmodulated RF); DAMPS, digital advance mobile phone system; FDMA, frequency division multiple access; FM, frequency modulated; GSM, global standard for mobile; PW, pulsed wave; TDMA, time division multiple access; UWB, ultra-wide band. Iridium is satellite telephony.

## References

[b1-ehp0115-000416] Adair RK (2003). Biophysical limits on athermal effects of RF and microwave radiation. Bioelectromagnetics.

[b2-ehp0115-000416] Adair ER Allen SJ Barber PW Guy AW Hurt WD Johnson CC et al. 1997. Radiofrequency Radiation Dosimetry Handbook, 4th ed. Brooks Air Force Base, TX:USAF School of Aerospace Medicine, Aerospace Medical Division (AFSC). Available: http://niremf.ifac.cnr.it/docs/HANDBOOK/contents.htm [accessed 7 February 2007].

[b3-ehp0115-000416] Advisory Group on Non-Ionizing Radiation 2003. Health Effects from Radiofrequency Electromagnetic Fields. Report of an Advisory Group on Non-ionising Radiation. Documents of the NRPB 14(2): Advisory Group on Non-Ionizing Radiation. Available: http://www.hpa.org.uk/radiation/publications/documents_of_nrpb/abstracts/absd14-2.htm [accessed 7 October 2006].

[b4-ehp0115-000416] Ahlbom A 2006. Studies on Base Stations and Other Telecommunications Towers. Available: http://www.who.int/peh-emf/meetings/archive/ahlbom_bsw.pdf [accessed 7 October 2006].

[b5-ehp0115-000416] Ahlbom A, Green A, Kheifets L, Savitz D, Swerdlow A, ICNIRP (International Commission for Non-Ionizing Radiation Protection) Standing Committee on Epidemiology. (2004). Epidemiology of health effects of radiofrequency exposure. Environ Health Perspect.

[b6-ehp0115-000416] American National Standards Institute/Institute of Electrical and Electronics Engineers 2006. IEEE Standard for Safety Levels with Respect to Human Exposure to Radio Frequency Electromagnetic Fields, 3 kHz to 300 GHz. C95.1-2005. Piscataway, NJ:American National Standards Institute/Institute of Electrical and Electronics Engineers, Inc.

[b7-ehp0115-000416] Anglesio L, Benedetto A, Bonino A, Colla D, Martire F, Saudino Fusette S (2001). Population exposure to electromagnetic fields generated by radio base stations: evaluation of the urban background by using provisional model and instrumental measurements. Radiat Prot Dosimetry.

[b8-ehp0115-000416] Aran JM, Carrere N, Chalan Y, Dulou PE, Larrieu S, Letenneur L (2004). Effects of exposure of the ear to GSM microwaves: *in vivo* and *in vitro* experimental studies. Int J Audiol.

[b9-ehp0115-000416] Ardoino L, Barbieri E, Vecchia P (2004). Determinants of exposure to electromagnetic fields from mobile phones. Radiat Prot Dosimetry.

[b10-ehp0115-000416] ARPANSA 2001. Regulatory Impact Statement: Radiation Protection Standard. Maximum Exposure Levels to Radiofrequency Fields—3 kHz to 300 GHz. Sydney:Australian Radiation Protection and Nuclear Safety Agency. Available: http://www.arpansa.gov.au/pubs/rps/ris.pdf [accessed 7 October 2006].

[b11-ehp0115-000416] ARPANSA 2002. Maximum Exposure Levels to Radiofrequency Fields—3 kHz to 300 GHz. Sydney:Australian Radiation Protection and Nuclear Safety Agency. Available: http://www.arpansa.gov.au/pubs/rps/rps3.pdf [accessed 7 October 2006].

[b12-ehp0115-000416] ARPANSA 2003a. What About Base Stations and Telecommunication Towers—Are There Any Health Effects? EME Fact Sheet no. 9. Sydney:Australian Radiation Protection and Nuclear Safety Agency. Available: http://www.arpansa.gov.au/pubs/eme_comitee/fact9.pdf [accessed 7 October 2006].

[b13-ehp0115-000416] ARPANSA 2003b. Mobile Phones and Children. Sydney: Australian Radiation Protection and Nuclear Safety Agency. Available: http://www.arpansa.gov.au/pubs/eme_comitee/fact11.pdf [accessed 7 October 2006].

[b14-ehp0115-000416] Barnett J Timotijevic L Shepherd R Senior V Vincent J 2006. Understanding Public Reactions to Precautionary Action and Advice. Available: http://www.who.int/peh-emf/meetings/archive/barnett_bsw.pdf [accessed 7 October 2006].

[b15-ehp0115-000416] Baumann J 2006. The Swiss Regulation and Its Application. Available: http://www.who.int/peh-emf/meetings/archive/baumann_bsw.pdf [accessed 7 October 2006].

[b16-ehp0115-000416] Berg G, Spallek J, Schuz J, Schlehofer B, Bohler E, Schlaefer K (2006). Occupational exposure to radio frequency/microwave radiation and the risk of brain tumors: interphone study group, Germany. Am J Epidemiol.

[b17-ehp0115-000416] Burch JB, Clark M, Yost MG, Fitzpatrick CT, Bachand AM, Ramaprasad J, Reif JS Radio frequency nonionizing radiation in a community exposed to radio and television broadcasting. Environ Health Perspect.

[b18-ehp0115-000416] Challis LJ (2005). Mechanisms for interaction between RF fields and biological tissue. Bioelectromagnetics.

[b19-ehp0115-000416] Christ A, Kuster N (2005). Differences in RF energy absorption in the heads of adults and children. Bioelectromagnetics.

[b20-ehp0115-000416] Coray R Krähenbühl P Reiderer M Stoll D Neubauer G 2002. Immissionen in Salzburg. Bundesamt für Metrologie und Akkreditierung, Lindenweg 50, CH-3003 Bern-Wabern. Available: http://www.6283.ch/docs/allgemein/Bakom/Salzburg_Bakom.pdf [accessed 7 February 2007].

[b21-ehp0115-000416] Cosquer B, Kuster N, Cassel JC (2005). Whole-body exposure to 2.45 GHz electromagnetic fields does not alter 12-arm radial-maze with reduced access to spatial cues in rats. Behav Brain Res.

[b22-ehp0115-000416] Dasenbrock C (2005). Animal carcinogenicity studies on radiofrequency fields related to mobile phones and base stations. Toxicol Appl Pharmacol.

[b23-ehp0115-000416] Durney CH Christensen DA 2000. Basic Introduction to Bioelectromagnetics. Boca Raton, FL:CRC Press.

[b24-ehp0115-000416] Ebert S, Eom SJ, Schuderer J, Apostel U, Tillmann T, Dasenbrock C (2005). Response, thermal regulatory threshold and thermal breakdown threshold of restrained RF-exposed mice at 905 MHz. Phys Med Biol.

[b25-ehp0115-000416] Elder JA, Chou CK (2003). Auditory response to pulsed radiofrequency energy. Bioelectromagnetics.

[b26-ehp0115-000416] Eltiti S, Wallace D, Zougkou K, Russo R, Joseph S, Rasor P, Fox E (2007). Development and evaluation of the electromagnetic hypersensitivity questionnaire. Bioelectromagnetics.

[b27-ehp0115-000416] Federal Communications Commission 2006. Information On Human Exposure To Radiofrequency Fields From Cellular and PCS Radio Transmitters. Available: http://www.fcc.gov/oet/rfsafety/cellpcs.html [accessed 7 October 2006].

[b28-ehp0115-000416] Feychting M (2005). Non-cancer EMF effects related to children. Bioelectromagnetics.

[b29-ehp0115-000416] Feychting M, Ahlbom A, Kheifets L (2005). EMF and health. Annu Rev Public Health.

[b30-ehp0115-000416] Foster K Radiofrequency exposure from wireless LANs utilizing Wi-Fi technology. Health Physics.

[b31-ehp0115-000416] Foster KR, Repacholi MH (2004). Biological effects of radiofrequency fields: does modulation matter?. Radiat Res.

[b32-ehp0115-000416] Fox E 2006. Health Effects of Mobile Phone Base-Stations: Human studies. Available: http://www.who.int/peh-emf/meetings/archive/fox_bsw.pdf [accessed 7 October 2006].

[b33-ehp0115-000416] Gotay CC (2005). Behavior and cancer prevention. J Clin Oncol.

[b34-ehp0115-000416] Health Canada 1999. Limits of Human Exposure to Radiofrequency Electromagnetic Fields in the Frequency Range from 3 KHZ to 300 GHZ - Safety Code 6. Available: http://www.hc-sc.gc.ca/ewh-semt/pubs/radiation/99ehd-dhm237/index_e.html [accessed 7 October 2006].

[b35-ehp0115-000416] Health Council. 2002. Mobile Telephones: An Evaluation of Health Effects. The Minister of Housing, Spatial Planning, and the Environment. Publication no 2002/01E. The Hague:Health Council of the Netherlands. Available: http://www.gr.nl/pdf.php?ID=377 [accessed 7 October 2006].

[b36-ehp0115-000416] Health Council. 2005. Electromagnetic Fields: Annual Update 2005. Publication no 2005/14. The Hague:Health Council of the Netherlands. Available: http://www.gr.nl/pdf.php?ID=1281&p=1 [accessed 7 October 2006].

[b37-ehp0115-000416] Health Physics Society 2006. Cellular Phones and Base Stations. Available: http://hps.org/publicinformation/ate/faqs/cellphoneqa.html [accessed 7 October 2006].

[b38-ehp0115-000416] Henderson BE, Ross RK, Pike MC (1991). Toward the primary prevention of cancer. Science.

[b39-ehp0115-000416] Hepworth SJ, Schoemaker MJ, Muir KR, Swerdlow AJ, van Tongeren MJ, McKinney PA (2006). Mobile phone use and risk of glioma in adults: case-control study. BMJ.

[b40-ehp0115-000416] ICNIRP (International Commission on Non-Ionizing Radiation Protection) 1998. Guidelines for Limiting Exposure to Time-Varying Electric, Magnetic, and Electromagnetic Fields. Available: http://www.icnirp.org/documents/emfgdl.pdf [accessed 7 October 2006].

[b41-ehp0115-000416] Independent Expert Group on Mobile Phones 2000. Mobile Phones and Health. Report of an Independent Expert Group on Mobile Phones. Available: http://www.iegmp.org.uk/ [accessed 7 October 2006].

[b42-ehp0115-000416] Jauchem JR (2003). A literature review of medical side effects from radio-frequency energy in the human environment: involving cancer, tumors, and problems of the central nervous system. J Microw Power Electromagn Energy.

[b43-ehp0115-000416] Kühn S Kramer A Lott U Kuster N 2006. Assessment of Human Exposure to Electromagnetic Radiation from Wireless Devices in Home and Office Environments. Available: http://www.who.int/peh-emf/meetings/archive/bsw_kuster.pdf [accessed 7 October 2006].

[b44-ehp0115-000416] Lahkola A Auvinen A Raitanen J Schoemaker MJ Christensen HC Feychting M 2007 Mobile phone use and risk of glioma in 5 North European countries Int J Cancer10.1002/ijc.22503 [Online 17January 2007]. 17230523

[b45-ehp0115-000416] Lonn S, Ahlbom A, Hall P, Feychting M, Swedish Interphone Study Group (2005). Long-term mobile phone use and brain tumor risk. Am J Epidemiol.

[b46-ehp0115-000416] Mann S Addison D Blackwell R Khalid M 2006. Laboratory and Volunteer Trials of a Personal RF Dosimeter. Available: http://www.who.int/peh-emf/meetings/archive/mann_bsw.pdf [accessed 7 October 2006].

[b47-ehp0115-000416] Markova E, Hillert L, Malmgren L, Persson BR, Belyaev IY (2005). Microwaves from GSM mobile telephones affect 53BP1 and gamma-H2AX foci in human lymphocytes from hypersensitive and healthy persons. Environ Health Perspect.

[b48-ehp0115-000416] McKean-Cowdin R, Feigelson HS, Ross RK, Pike MC, Henderson BE (2000). Declining cancer rates in the 1990s. J Clin Oncol.

[b49-ehp0115-000416] Mild KH Karlstrom EF Hamberg L Tornevik C 2006. Occupational RF Exposure from Base Station Antennas on Roof-Tops and Buildings. Available: http://www.who.int/peh-emf/meetings/archive/hanssonmild_bsw.pdf [accessed 7 October 2006].

[b50-ehp0115-000416] National Council for Radiation Protection 2003. Biological Effects of Modulated Radiofrequency Fields. Commentary No. 18. Bethesda, MD:National Council for Radiation Protection. Available: http://www.ncrppublications.org/index.cfm?fm=Product.AddToCart&pid=4191384437 [accessed 7 October 2006].

[b51-ehp0115-000416] Nelson PD, Toledano MB, McConville J, Quinn MJ, Cooper N, Elliott P (2006). Trends in acoustic neuroma and cellular phones: is there a link?. Neurology.

[b52-ehp0115-000416] NRPB/HPA (National Radiation Protection Board/Health Protection Agency) 2004a. Review of the Scientific Evidence for Limiting Exposure to Electromagnetic Fields (0–300 GHz). Available: http://www.hpa.org.uk/radiation/publications/documents_of_nrpb/pdfs/doc_15_3.pdf [accessed 7 October 2006].

[b53-ehp0115-000416] NRPB/HPA (National Radiation Protection Board/Health Protection Agency) 2004b. Mobile Phones and Health 2004. Report by the Board of NRPB. Available: http://www.hpa.org.uk/radiation/publications/documents_of_nrpb/abstracts/absd15-5.htm [accessed 7 October 2006].

[b54-ehp0115-000416] Parkinson WC (1985). Electromagnetic fields in biological studies. Ann Biomed Eng.

[b55-ehp0115-000416] Polk C Postow E eds. 1996. Handbook of Biological Effects of Electromagnetic Fields. Boca Raton, FL:CRC Press.

[b56-ehp0115-000416] Renn O (2004). Perception of risks. Toxicol Lett.

[b57-ehp0115-000416] Renn O 2006. Risk Communication about EMF: Insights and Challenges. Available: http://www.who.int/peh-emf/meetings/archive/renn_bsw.pdf [accessed 7 October 2006].

[b58-ehp0115-000416] Repacholi MH (1998). Low-level exposure to radiofrequency electromagnetic fields: health effects and research needs. Bioelectromagnetics.

[b59-ehp0115-000416] Royal Society of Canada 2004. Recent Advances in Research on Radiofrequency Fields and Health: 2001–2003: A Follow-up to The Royal Society of Canada Report on the Potential Health Risks of Radiofrequency Fields from Wireless Telecommunication Devices (Krewski D, Byus CV, Glickman BW, Habash RWY, Habbick B, Lotz WG, et al.). Ottawa:Royal Society of Canada. Available: http://www.rsc.ca//files/publications/expert_panels/RF//expert_panel_radiofrequency_update2.pdf [accessed 7 October 2006].

[b60-ehp0115-000416] Rubin GJ, Das Munshi J, Wessely S (2005). Electromagnetic hypersensitivity: a systematic review of provocation studies. Psychosom Med.

[b61-ehp0115-000416] Rubin GJ, Das Munshi J, Wessely S (2006a). A systematic review of treatments for electromagnetic hypersensitivity. Psychother Psychosom.

[b62-ehp0115-000416] Rubin GJ, Hahn G, Everitt BS, Cleare AJ, Wessely S (2006b). Are some people sensitive to mobile phone signals? Within participants double blind randomised provocation study. BMJ.

[b63-ehp0115-000416] Schoemaker MJ, Swerdlow AJ, Ahlbom A, Auvinen A, Blaasaas KG, Cardis E (2005). Mobile phone use and risk of acoustic neuroma: results of the Interphone case-control study in five North European countries. Br J Cancer.

[b64-ehp0115-000416] Schuz J, Bohler E, Berg G, Schlehofer B, Hettinger I, Schlaefer K (2006). Cellular phones, cordless phones, and the risks of glioma and meningioma (INTERPHONE study group, Germany). Am J Epidemiol.

[b65-ehp0115-000416] Seitz H, Stinner D, Eikmann T, Herr C, Roosli M (2005). Electromagnetic hypersensitivity (EHS) and subjective health complaints associated with electromagnetic fields of mobile phone communication—a literature review published between 2000 and 2004. Sci Total Environ.

[b66-ehp0115-000416] Siegrist M, Earle TC, Gutscher H, Keller C (2005). Perception of mobile phone and base station risks. Risk Anal.

[b67-ehp0115-000416] Takebayashi T, Akiba S, Kikuchi Y, Taki M, Wake K, Watanabe S (2006). Mobile phone use and acoustic neuroma risk in Japan. Occup Environ Med.

[b68-ehp0115-000416] Tell RA, Mantiply ED (1980). Population exposure to VHF and UHF broadcast radiation in the United States. Proc IEEE.

[b69-ehp0115-000416] Titushkin I, Cho M (2006). Distinct membrane mechanical properties of human mesenchymal stem cells determined using laser optical tweezers. Biophys J.

[b70-ehp0115-000416] Valberg PA 2006. Modulated RF Energy: Mechanistic Viewpoint on the Health Implications. Available: http://www.who.int/peh-emf/meetings/archive/valberg_bsw.pdf [accessed 7 October 2006].

[b71-ehp0115-000416] Vangelova K, Deyanov C, Israel M (2006). Cardiovascular risk in operators under radiofrequency electromagnetic radiation. Int J Hyg Environ Health.

[b72-ehp0115-000416] Vecchia P 2006. Base Stations and Health: Government Responses in Italy. Available: http://www.who.int/peh-emf/meetings/archive/vecchia_lecture_bsw.pdf [accessed 7 October 2006].

[b73-ehp0115-000416] Veyret B 2006. A Review of Non-thermal Health Effects from RF Fields. Available: http://www.who.int/peh-emf/meetings/archive/veyret_bsw.pdf [accessed 7 October 2006].

[b74-ehp0115-000416] Vijayalaxmi, Obe G (2004). Controversial cytogenetic observations in mammalian somatic cells exposed to radiofrequency radiation. Radiat Res.

[b75-ehp0115-000416] WHO 2005. Electromagnetic Hypersensitivity. Geneva:World Health Organization. Available: http://www.who.int/mediacentre/factsheets/fs296/en/ [accessed 7 October 2006].

[b76-ehp0115-000416] WHO 2006a. WHO EMF Research Databases. Geneva:World Health Organization. Available: http://www.who.int/peh-emf/research/database/en/ [accessed 7 October 2006].

[b77-ehp0115-000416] WHO 2006b. WHO 2006 Research Agenda for Electromagnetic Fields. Geneva:World Health Organization. Available: http://www.who.int/peh-emf/research/rf_research_agenda_2006.pdf [accessed 7 October 2006].

[b78-ehp0115-000416] Wiedemann PM, Schutz H (2005). The precautionary principle and risk perception: experimental studies in the EMF area. Environ Health Perspect.

[b79-ehp0115-000416] Wilen J, Hornsten R, Sandstrom M, Bjerle P, Wiklund U, Stensson O (2004). Electromagnetic field exposure and health among RF plastic sealer operators. Bioelectromagnetics.

[b80-ehp0115-000416] Wogan GN, Hecht SS, Felton JS, Conney AH, Loeb LA (2004). Environmental and chemical carcinogenesis. Semin Cancer Biol.

